# Effects of Levels of Self-Regulation and Regulatory Teaching on Strategies for Coping With Academic Stress in Undergraduate Students

**DOI:** 10.3389/fpsyg.2020.00022

**Published:** 2020-01-31

**Authors:** Jesús de la Fuente, Jorge Amate, María Carmen González-Torres, Raquel Artuch, Juan Manuel García-Torrecillas, Salvatore Fadda

**Affiliations:** ^1^School of Education and Psychology, University of Navarra, Pamplona, Spain; ^2^School of Psychology, University of Almería, Almería, Spain; ^3^Provincial Educational Authority, Almería, Spain; ^4^School of Education, International University of La Rioja, Logroño, Spain; ^5^Research Unit, Torrecardenas Hospital, Almería, Spain; ^6^Instituto de Investigación Biosanitaria ibs, Granada, Spain; ^7^Centro de Investigación Biomédica en Red de Epidemiología y Salud Pública, Madrid, Spain; ^8^Prevention Service, University of Sassari, Sassari, Italy

**Keywords:** SRL vs. ERL theory, academic stress, coping strategies, university, students

## Abstract

The *SRL vs. ERL Theory^TM^* predicts that regulation-related factors in the student and in the context combine to determine the student’s levels in emotional variables, stress, and coping strategies. The objective of the present research was to test this prediction in the aspect of coping strategies. Our hypothesis posed that students’ level of self-regulation (low–medium–high), in combination with the level of regulation promoted in teaching (low–medium–high), would determine the type of strategies students used to cope with academic stress; the interaction of these levels would focus coping strategies either toward emotions or toward the problem. A total of 944 university students completed validated questionnaires on self-regulation, regulatory teaching, and coping strategies, using an online tool. ANOVAs and MANOVAs (3 × 1; 3 × 3; 5 × 1) were carried out, in a quasi-experimental design by selection. Level of self-regulation and level of regulatory teaching both had a significant effect on the type of coping strategies used. The most important finding was that the combined level of self-regulation and external regulation, on a five-level scale or heuristic, predicted the type of coping strategies that were used. In conclusion, the fact that this combination can predict type of coping strategies used by the student lends empirical support to the initial theory. Implications for the teaching–learning process at university and for students’ emotional health are discussed.

## Introduction

The study of students’ emotional experiences in the teaching and learning context has yielded much research on aspects previously unconsidered by the cognitivist paradigm (Linnenbrink-Garcia and Pekrun, 2011; Goetz et al., 2014; Lüftenegger et al., 2016; Murayama et al., 2017; Duffy et al., 2018; Gentsch et al., 2018; Loderer et al., 2018; Collie et al., 2019; Harley et al., 2019; Hirvonena et al., 2019). In the present study, our effort has focused on explaining the degree to which emotional processes facilitate or interfere in cognitive processes (Rusk et al., 2011; Moffa et al., 2016; Putwain, 2018).

### Academic Stress at University

In the university context, due to the difficulty of meeting the demands and requirements of study, the experience of stress is an important phenomenon that has captured research interest (Martín et al., 2003; Cabanach et al., 2007; Willcoxson et al., 2011; D’Mello, 2013; Pidgeon and Pickett, 2017; Scharp and Dorrance, 2017). Research on *academic stress* in this context, from the perspective of Clinical and Health Psychology, has prioritized individual predictive or explanatory factors, with particular focus on differentiating factors like personality variables, anxiety, or cognitive differences (Palmer and Rodger, 2009; Saklofske et al., 2012; Dicke et al., 2018; Cassady et al., 2019). From an Educational Psychology perspective, however, it seems reasonable to approach the study of stress as a contextualized phenomenon within the teaching-learning process (Mainhard et al., 2018). On one hand, the learning process can by accompanied by the experience of stress and by the use of resources for managing stress (coping strategies), depending on characteristics of the individual (Shaw et al., 2017; Rapillard et al., 2019). On the other hand, the context, or teaching process, can give rise to stressful experiences and to the use of stress management methods (Frenzel et al., 2018; Gentsch et al., 2018; Collie et al., 2019). The present research report adopts an interactive student-teacher approach to academic stress, analyzing stress that arises from the interaction of the student’s learning process with characteristics of the teaching process.

### Coping Strategies as a Variable of the Teaching and Learning Process

Coping strategies are a psychological construction referring to knowledge, skills and strategic behaviors that people use to manage emotions occurring within a situation of stress (Fimian et al., 1989; Chartier et al., 2011; Freire et al., 2018); for this reason, they are considered meta-emotional skills (de la Fuente et al., 2017a). Multiple models have been proposed for categorizing these skills, beginning with the initial model proposed by Lazarus and Folkman (1984/1986) and Lazarus (1999). In essence, two types of strategies have been described: (1) those that seek to minimize negative emotional states, i.e., *emotion-focused strategies;* and (2) those that address the cause of the stressful experience or of overtaxed personal resources, i.e., *problem-focused strategies*. In the initial research it was assumed that stress was associated only with negative emotionality; however, the reformulated versions of the theoretical model assumed that it was possible to combine mixed coping strategies (Folkman, 1997, 2008, 2011).

#### Coping Strategies in the Learning Process

Prior research on motivational and affective factors of learning in university contexts has also recognized the importance of the different types of coping strategies used by university students. Some examples have addressed the role of religious coping (Francis et al., 2018), the role of health habits as a coping strategy (Tada, 2017), how coping strategies related to well-being (Park and Adler, 2003; Bhullar et al., 2014; Freire et al., 2016), types of coping and their relationship to resilience, academic coping within a religious vs. secular context (González-Torres and Artuch, 2014). The associations between coping strategies, anxiety and engagement-burnout have also been established (de la Fuente et al., 2015a).

#### Coping Strategies in the Teaching Process

Some prior research has analyzed *coping strategies* from the teacher’s standpoint: their methods of coping (Gustems-Carnicer et al., 2019), and their levels of stress (Browers and Tomic, 2000; Alson, 2019). From a complementary viewpoint, teacher effectiveness at university has been measured in terms of students’ well-being and good teacher-student relations (Lekwa et al., 2018; Aldrupa et al., 2019). Evidence has also shown the influence of teachers’ personality characteristics in effective teaching (Kim et al., 2019).

#### Combined Effect of Teaching and Learning Process Variables on Coping Strategies

However, the effect of this combination on types of coping strategies used by university students, as a consequence of the teaching and learning process, has not been sufficiently established (de la Fuente et al., 2016, 2017b). The present research, therefore, focuses on how combined levels of *Student Self-Regulation* (SR) (learning process) and *Teaching Effectiveness* (teaching process) determine types of coping strategies in students. This research report is part of a series of complementary papers that present evidence of the combined effects of these two types of variables on students’ emotional variables (de la Fuente et al., 2019).

### SRL vs. ERL Theory as a Research Heuristic in the Teaching and Learning Process

The theory of *Self- vs. Externally- Regulated Learning* is founded conceptually on the assumptions below (see de la Fuente, 2017). It is a further development of the concept of self-regulated learning from B. J. Zimmerman’s model (Zimmerman, 2001, 2008; Zimmerman and Labuhn, 2012) and of Vermunt’s concept of self-regulation and external regulation (Vermunt, 1998, 2005; Vermunt and Vermetten, 2004; Vermunt et al., 2014; Vermunt and Donche, 2017). The theoretical model of SRL vs. ERL defines different types of regulation along a behavioral continuum. This continuum is useful for analyzing the *teaching and learning process*:

(1) With regard to the *learning process*, the model defines three levels of student regulation in a learning situation:
  *Self-Regulation* represents a *high degree of self-regulation* or *positive proactivity*, that is, active and adequate regulation of one’s own behavior (level 3 of SR).
  *Non-Regulation* (NR) refers to a lack of proactivity or *a medium level of self-regulation*. This is the conceptual equivalent of *reactivity* (level 2 of SR).
  *Dysregulation* (DR) is negative proactivity or a *low level of self-regulation.* The individual actively manages his or her own behavior toward inadequate purposes (level 1 of SR).
  In summary, level of SR, as a personal characteristic of the student, predisposes an equivalent level of self-regulated learning (Zimmerman, 2001, 2008; Zimmerman and Labuhn, 2012).
(2) With regard to the *teaching process*, this model defines several levels of regulatory teaching (RT), or levels of teaching effectiveness. The present model is more explicit than Zimmerman’s SRL model (Zimmerman, 2001, 2008; Zimmerman and Labuhn, 2012), since it specifically defines the value of each level of teaching effectiveness for predisposing self-regulated learning, an aspect not clearly defined in the previous model.
  *Externally Regulatory (ER) teaching* or *highly effective teaching*. In this context, the teaching prompts students toward well-directed proactivity and SR. This type of teaching context provides many external indicators that increase the likelihood of self-regulated behavior (before, during and after) (Level 3 RT).
  *Externally Non-regulatory* (ENR) *teaching* or *moderately effective teaching.* Whether at the beginning, middle or end of learning acts, there are no external indicators or promptings that encourage self-regulated or dysregulated behavior, or that increase the likelihood of one or the other. A non-regulatory context requires the student to engage in a moderate level of self-regulated behavior, given that contextual elements offer no direction (Level 2 RT).
  *Externally Dys-Regulatory* (EDR) *teaching* or *ineffective teaching*. Dysregulation, that is, inadequate or negative proactivity, is actively promoted in this context. The individual who wishes to practice self-regulated learning in this type of context must make a great effort (Level 1 RT).
(3) Effects of the *combined levels of self-regulation and external regulation* can be predicted. Human learning takes its shape when the individual’s self-regulating ability (SR) and the external regulatory features of the context (ER) are combined. Five types of interactions are possible (de la Fuente et al., 2019). According to this principle, coping strategies are predisposed by mediating factors, both internal (self-regulation, SR: levels 1–3) and external (external regulation, ER: levels 1–3). This theoretical model requires that *subject x context* interactions be specified, addressing an insufficiency of the initial theoretical model of Self-Regulated Learning (Zimmerman, 2001, 2008; Zimmerman and Labuhn, 2012).

### Aims and Hypothesis

Based on the models and previous empirical data, the following objectives were identified: (1) to establish whether the university students’ personal regulation levels and the regulatory levels of their context, independently of each other, determined the type of coping strategies used; (2) to establish whether the combined levels of SR and RT, as described in the interactivity model proposed above, were associated with the type of coping strategies used. Based on these objectives, the *hypothesis* established that a *graded increase in level of regulation* (internal and external) would give rise to (1) a proportionate decrease in emotion-focused strategies, and (2) a proportionate increase in problem-focused coping strategies. By contrast, a *graded decrease in level of regulation* (internal and external) would give rise to (1) a proportionate increase in emotion-focused strategies, and (2) a proportionate decrease in problem-focused coping strategies.

## Materials and Methods

### Participants

To establish interdependence relations among low-medium-high levels of SR, and RT, we used a total sample of 944 undergraduate students from two universities of Spain. The sample was composed of students enrolled in Psychology, Primary Education, and Early Childhood Education degrees; 82.7% were women and 17.3% were men. Their ages ranged from 19 to 45, with a mean age of 22.25 (σ_*X*_ = 6.3) years. Of the total sample, 28.3% were first-year students, 40.3% were in second year, 14.5% in third year, and 16.5% were in the fourth year of the degree program.

### Instruments

#### Self-Regulation

This variable was measured using the *Short Self-Regulation Questionnaire (SSRQ)* (Miller and Brown, 1991). Previously validated in Spanish samples (Pichardo et al., 2014, 2018), it possesses acceptable validity and reliability values, similar to the English version. The original SRQ (Brown et al., 1999) evaluates subjects’ SR of behavior, understood as the ability to plan and manage their own behavior in a flexible way, according to the desired outcomes. Although the questionnaire has been adapted to educational contexts, it was initially designed within the field of addictive behaviors. The authors, using squared multiple correlation coefficients, carried out an initial design of 63 items (26 reverse) that constituted 7 scales: (1) informational input, which refers to a person’s ability to obtain information on their current state from their environment; (2) self-evaluation, where this information is compared to personal goals, rules and expectations; (3) instigation to change, the person’s perception of any existing discrepancies between their current state and their desired state; (4) search for ways to reduce discrepancies; (5) planning for change, that is, strategies or actions for carrying out the change process; (6) implementation of the change strategies; and (7) evaluation of progress toward a goal. The English version of the instrument has mainly been used with university students. Different studies have analyzed the SRQ’s psychometric properties, establishing several factorial solutions. Carey et al. (2004), using a sample of 391 American undergraduate students between the ages of 17 and 24, established a one-factor solution composed of 31 items, which led the authors to propose a new measure: the Short SRQ (SSRQ). Correlation between the two versions was strong (*r* = 0.96), suggesting that the short version is a good alternative to the full scale.

The *Short SRQ* is composed of four factors (goal setting-planning, perseverance, decision making and learning from mistakes) and 17 items (all of them with saturations greater than 0.40); the confirmatory factor structure is consistent (χ^2^ = 250.83, *df* = 112, CFI = 0.90, GFI = 0.92, AGFI = 0.90, RMSEA = 0.05). *Internal consistency* was acceptable for the total of questionnaire items (α = 0.86) and for the factors of goal setting-planning (α = 0.79; six items), decision making (α = 0.72; three items) learning from mistakes (α = 0.72; five items), and perseverance (α = 0.73; three items). *Correlations* have been studied between each item and its factor total, between the factors, and between each factor and the complete questionnaire, with good results for all, except for the decision-making factor, which showed a weaker correlation with other factors (range: 0.41–0.58). Correlations of the long and short Spanish versions (long SRQ with 32 items and short SRQ with 17 items), to the original long questionnaire, are better for the short version (short Spanish to long English questionnaire: *r* = 0.85 and short Spanish to long Spanish: *r* = 0.94; *p* < 0.01) than for the long Spanish version (long Spanish to long English: *r* = 0.79; *p* < 0.01). For more information, please, see: https://www.frontiersin.org/articles/10.3389/fpsyg.2019.01919/full#supplementary-material.

#### Regulatory Teaching (Teaching Effectiveness)

The *Scales for Assessment of the Teaching-Learning Process, ATLP, student version* (de la Fuente et al., 2012) were used to evaluate students’ perception of the teaching process. The scale entitled *Regulatory Teaching* is Dimension 1 of the confirmatory model. ATLP-D1 comprises 29 items structured along five factors: Specific RT, regulatory assessment, preparation for learning, satisfaction with the teaching, and general RT. The scale was validated in university students (de la Fuente et al., 2012) and showed a factor structure with adequate fit indices (χ^2^ = 590.626; *df* = 48, *p* < 0.001, CF1 = 0.938, TLI = 0.939, NFI = 0.950, NNFI = 0.967; RMSEA = 0.068) and adequate internal consistency (ATLP D1: α = 0.83; Specific RT, α = 0.897; regulatory assessment, α = 0.883; preparation for learning, α = 0.849; satisfaction with the teaching, α = 0.883 and general RT, α = 0.883). The ATLP is a self-report instrument to be completed by the teacher and the students, available in Spanish and English versions. It also includes a qualitative part where students can make recommendations for improving each of the processes evaluated. As for external validity, results are also consistent, since there are different interdependent relationships among perceptions of variables that exist in an academic environment. For more information, please, see: https://www.frontiersin.org/articles/10.3389/fpsyg.2019.01919/full#supplementary-material.

#### Coping Strategies

The *Coping Strategies Scale*, EEC (Chorot and Sandín, 1987) was used, in a short validated Spanish version, EEC-Short (de la Fuente, 2014). Although the original instrument contained 90 items, the validation produced a first-order structure of 64 items and a second order with 10 factors and two dimensions, both of them significant, with adequate fit values in the latter [χ^2^ = 878,75; *df*(77-34) = 43, *p* < 0.001; NFI = 0.901; RFI = 0.945; IFI = 0.903; TLI = 0.951; CFI = 0.903, RMSEA = 0.07]. Reliability measures are Cronbach alpha of 0.93 (complete scale), 0.93 (first half) and 0.90 (second half), Spearman–Brown of 0.84 and Guttman of 0.80. The scale assesses two dimensions: D1: Emotion-focused coping (0.95) and D2: Problem-focused coping (0.91). The emotion-focused strategies were: F1. Avoidant distraction (0.79); F7. Reducing anxiety and avoidance (0.88); F8. Preparing for the worst (0.80); F9. Emotional venting and isolation (0.91); and F11. Resigned acceptance (0.86). The problem-focused strategies were: F2. Seeking help and counsel (0.92); F5. Self-instructions (0.82); F10. Positive reappraisal and firmness (0.87); F12. Communicating feelings and social support (0.89); and F13. Seeking alternative reinforcement (0.80). See Table 1.

**TABLE 1:** Types of coping strategies and examples of items in the short EEC version.

***Emotion-focused coping* (D1)**	***Example of ítems***
F1. Avoidant distraction	I get away and forget the problem temporarily (change of environment)
F7. Reducing anxiety and avoidance	I practice some kind of sport in order to reduce my anxiety or tension
F8. Preparing for the worst	I prepare myself for the worst
F9. Emotional venting and isolation	I act irritable and aggressive toward others
F11. Resigned acceptance	I accept the problem as it is, since I cannot do anything about it
***Problem-focused coping* (D2)**	
F2. Seeking help and counsel	I talk with people I know who can do something to solve my problem
F5. Self-instructions	I set out a plan of action and try to carry it out
F10. Positive re-appraisal and firmness	I try to see positive aspects of the situation
F12. Comunicating feelings and social support	I feel better if I explain my problem to friends or family members
F13. Seeking alternative reinforcement	I start new activities (studies, etc.)

### Procedure

Participants voluntarily completed the scales using an online *platform* (de la Fuente et al., 2015b). A total of ten specific teaching-learning processes were evaluated, covering different university subjects that were taught within a 2-year period. Based on Biggs’ 3P model (Biggs, 2001), *Presage* variables (SR) were assessed in September-October of 2017 and 2018; *Process* variables (Coping Strategies) and *Product* variables (RT) were assessed in May-June of 2017 and 2018. The students self-reported on: (1) self-regulation characteristics (SR) at the beginning of the academic year; (2) coping strategies (CS) and RT at the end of the course. Each group of students only evaluated one teaching-learning process. The procedure was approved by the respective Ethics Committees of each university, in the context of two R&D Projects (2018–2021).

### Data Analysis

#### Effects of Regulation Levels

Through cluster analysis, continuous independent variables were transformed into discrete dependent variables with three levels (low-medium-high). Using an *ex post*-facto design, a 3 K-means cluster analysis was first conducted to establish low-medium-high groups in the two variables: Personal SR and RT. For the SR variable, values of Low = 2.70; Medium = 3.48; High = 4.20 formed the centers of the clusters, and ranges were as follows: low, 1.00–3.09; medium, 3.10–3.84; and high, 3.85–5.00. For the RT variable, Low = 2.72; Medium = 3.58; High = 4.34 formed the centers of the clusters, and ranges were: Low, 1.00–2.34; Medium, 2.35–2.83; and High, 2.84–5.00. In addition, several ANOVAs and MANOVAs were carried out, in order to ascertain the effect of low-medium-high levels on the dependent variable, coping strategies. Also, using a 3-factor design (low-medium-high SR levels) × 3 (low-medium-high levels of RT), several MANOVAs were conducted, taking these levels as independent variables. Finally, based on the low–medium–high groups in both variables (SR and RT), five combinations were configured, according to the proposed theoretical model (see Table 2). MANOVAs were conducted to establish statistical suitability of these groupings, as well as the effects on the defined dependent variables, with Pillai’s trace and Sheffé test index.

**TABLE 2:** Combinations between parameters of the model hypothesized in SRL vs. ERL Theory: the *Utility Model^TM^* (de la Fuente et al., 2019, p. 12).

***Combination Levels***		***Regulation mean/rank***	***Regulation Range***	***Emotions Stress***			***Coping Facors* and *Effect***	Strateg.*
**SR Level (range)**	**RT Level (range)**			**>**		**<**		
**3** (3.85 – 5.00) **H**	**3** (2.84 – 5.00) **H**	3.0/**5**	**High-High:** *High Regulation*	++		*−*	*Low*	+*Pr/−Em*
**2** (3.10 – 3.84) **M**	**3** (2.84 – 5.00) **H**	2.5/**4**	**Medium-High**: *Regulation*	+		*−*	*M-L*	+*Pr/−Em*
**3** (3.85 – 5.00) **H**	**2** (2.35 – 2.83) **M**	2.5/**4**	**High-Medium**: *Regulation*	+		*−*	*M-L*	+*Pr/−Em*
**2** (3.10 – 3.84) **M**	**2** (2.35 – 2.83) **M**	2.0/**3**	**Medium:** *Non-regulation*	+		*−*	*M*	*=Pr/ = Em*
**2**(3.10 – 3.84) **M**	**1** (1.00 – 2.34) **L**	1.5/**2**	**Medium-Low**: *Dysregulation*	*−*	=	+	*M-H*	+*Em/−Pr*
**1** (1.00 – 3.09) **L**	**2** (2.35 – 2.83) **M**	1.5/**2**	**Low-Medium**: *Dysregulation*	*−*		+	*M-H*	+*Em/−Pr*
**1** (1.00 – 3.09) **L**	**1** (1.00 – 2.34) **L**	1.0/**1**	**Low-Low:** *High Dysregulation*	*− −*		+	*High*	+*Em/−Pr*

H, High; M, Medium; L, Low; *Emotions*: + (positives) vs. *−* (negatives). *Dependent Variable in this study: *Coping Strategies: Pr*, Problem-focused Coping; Em, Emotion-focused Coping.

#### A Combination Typology for Understanding Coping Strategies

The multivariate analyses (MANOVAs) showed a statistically significant main effect of the five combination types of low-medium-high levels of SR and RT (see de la Fuente et al., 2019, p.12, and Table 2):

*Combination 1* presented a statistically significant low level in *SR* and low level in *RT (1 and 1)*. The **average of regulation levels is 1.0**, and the **rank** is **1**. The regulation range is low SR and low RT, associated with a *low level of self-regulation* or *high level of dysregulation*. Consequently, the effects are a *high level of emotion-focused coping strategies and a low level of problem-focused coping strategies.*

*Combination 2* had a statistically significant low level in *SR* and medium level in *RT*, or vice versa *(2 and 1, or 1 and 2).* The **average of regulation levels is 1.5,** and the **rank** is **2.** The regulation range is low-medium SR and low-medium RT, and vice versa, associated with a *medium-low level of self-regulation or medium-high level of dys-regulation*. Consequently, the effects are a *medium-high level of emotion-focused coping strategies and medium-low levels of problem-focused coping strategies.*

*Combination 3* presented a statistically significant medium SR level and medium RT level *(2 and 2).* The **average of regulation levels is 2.0,** and the **rank** is **3**. The regulation range is medium SR and medium RT, associated with a *medium level of self-regulation or dys-regulation*. Consequently, the effects are a *medium level of emotion-focused coping strategies* and *medium level of problem-focused coping strategies.*

*Combination 4* had a statistically significant medium SR with high RT or high SR with medium RT (*2 and 3*, or *3 and 2*). The **average of regulation levels is 2.5**, and the **rank** is **4.** The regulation range is high SR-medium RT, or medium SR-high RT, associated with a medium-high level of *self-regulation* or medium-low level of *dys-regulation*. Consequently, the effects are a *medium-low level of emotion-focused coping strategies and medium-high level of problem-focused coping strategies.*

*Combination 5* presented a statistically significant high SR and high RT *(3 and 3).* The **average of regulation levels is 3.0**, and the **rank** is **5.** The regulation range is high SR-high RT, associated with a *high level* of *self-regulation* and low level of *dys-regulation*. Consequently, the effects are a *low level of emotion-focused coping strategies* and *high level of problem-focused coping strategies.*

## Results

### Interdependent Simple Effects of Levels of Personal Self-Regulation (SR) and Levels of Regulatory Teaching (RT) on Stress Coping Strategies

#### Effect of Self-Regulation on Stress Coping Strategies

A statistically significant effect was noted of the *IV SR* (low-medium levels) on total *Coping Strategies.* The statistically significant partial effect of the *IV SR* (low-medium-high levels) was maintained for the two dimensions of *Emotion-focused Coping Strategies* and *Problem-focused Coping Strategies*, the latter showing greater statistical significance.

A statistically significant main effect of the *IV SR* (low-medium-high levels) was noted on the factors of *Emotion-focused Coping Strategies*. Also, the statistically significant partial effect of the *IV SR* (low-medium-high levels) was maintained for *F1* (Avoidant distraction), *F7* (Reducing anxiety and avoidance), *F8* (Preparing for the worst), with greater statistical significance for factors *F9* (Emotional venting and isolation) and *F11* (Resigned acceptance), for university students with lower levels of SR. Complementarily, a statistically significant main effect of the *IV SR* (low–medium–high levels), was noted on the factors of *Problem-focused Coping Strategies*. Also, the statistically significant partial effect of the *IV SR* (low-medium-high levels) was maintained for *F2* (Seeking help), *F5* (Self-Instructions), *F10* (Positive re-appraisal and firmness), *F12* (Communicating feelings and social support), *F13* (Seeking alternative reinforcement). See Table 3.

**TABLE 3:** Interdependence relations between low–medium–high levels of *Self-Regulation (SR)* and *Regulatory Teaching (RT)* as independent variables, in strategies for coping with stress.

***DVs***	***Self-Regulation (SR)***				***Effects***
	***1. Low***	***2.Medium***	***3. High***	***Average***	
	**(*n* = 240)**	**(*n* = 429)**	**(*n* = 275)**	**(*n* = 944)**	
Coping Strategies					
*Total*	2.66 (0.28)	2.66 (0.26)	2.71(0.28)*	2.67 (0.27)	*F* (2, 941) = 3.265 (Pillai’s), *p* < 0.05; *n^2^* = 0.007, pw = 0.622

*Dimensions*					*F*(4,1882) = 40.770 (Pillai’s), *p < 0.00l, n^2^ = 0.080*, pw = 1.0
D1. Emotion-focused	2.51(0.34)*	2.43 (0.30)	2.37 (0.32)	2.43 (0.32)	*F*(2,941) = 12.892, *p <* 0.001, *n^2^* = 0.026, 1 > 2 > 3
D2. Problem-focused	2.80 (0.34)	2.89 (0.31)	3.00(0.33)*	2.92 (0.75)	*F*(2,941) = 38.765, *p <* 0.001, *n^2^* = 0.076, 1 < 2 < 3*

*Emotion-focused strategies (factors)*					*F*(10,1858) = 21.011 (Pillai’s), *p* < 0.001, n2 = 0.107
F1. Avoidant distraction	2.33(0.51)*	2.27 (0.48)	2.20 (0.51)	2.26 (0.50)	*F*(2,1056) = 4.431, *p* < 0.01, *n^2^* = 0.008; 1 > 3
F7. Reducing anxiety	3.11(0.64)*	3.05 (0.59)	2.91 (0.69)	3.02 (0.64)	*F*(2,1056) = 7.954, *p* < 0.001, *n^2^* = 0.015, 1 > 2 > 3
F8. Preparing for the worst	2.83(0.47)*	2.66 (0.46)	2.56 (0.46)	2.67 (0.47)	*F*(2,1056) = 24.302, *p* < 0.001, *n^2^* = 0.044; 1 > 2 > 3
F9. Emotional venting	2.09(0.48)*	1.90 (0.42)	1.68 (0.37)	2.67 (0.47)	*F*(2,1056) = 68.259, *p* < 0.001, *n^2^* = 0.114; 1 > 2 > 3*
F11. Resigned acceptance	2.29(0.56)*	2.05 (0.47)	1.78 (0.48)	2.04 (0.53)	*F*(2,1056) = 74.507, *p* < 0.001, *n^2^* = 0.124; 1 > 2 > 3*

*Problem-focused strategies (factors)*					*F*(10,2132) = 19391 (Pillai’s), *p* < 0.001, *n^2^* = *0.125*
F2. Seeking help	2.80 (0.73)	2.95 (0.65)	3.05(0.66)*	2.87 (0.86)	*F*(2,1069) = 9,713 (Pillai’s), *p* < 0.001, *n^2^* = 0.018; 3 > 2 > 1
F5. Self-Instructions	2.86 (0.44)	3.05 (0.88)	3.07 (0.43)	3.29(0.39)*	*F*(2,1069) = 86.880, *p* < 0.001, *n^2^* = 0.125; 3 > 2 > 1*
F10. Positive re-appraisal	2.77 (0.49)	3.06 (0.42)	3.05 (0.73)	3.39(0.39)*	*F*(2,1069) = 144.769, *p* < 0.001, *n^2^* = 0.213; 3 > 2 > 1*
F12. Communicating feelings	2.90 (0.79)	3.05 (0.71)	3.17(0.70)*	2.57 (0.94)	*F*(2,1069) = 9.706, *p* < 0.001, *n^2^* = 018; 3 > 2 > 1
F13. Alternative reinforcement	2.79 (0.40)	2.81 (0.41)	2.93(0.45)*	2.84 (0.43)	*F*(2,1069) = 9.486, *p* < 0.001, *n^2^* = 0.017; 3 > 2,1

***DVs***	***Regulatory Teaching (RT)***				
	***1. Low***	***2. Medium***	***3. High***	***average***	
	**(*n* = 159)**	**(*n* = 390)**	**(*n* = 293)**	**(*n* = 842)**	

***Coping Strategies***					
*Total*	2.60 (0.28)	2.63 (0.25)	2.74(0.28)*	2.66 (0.78)	*F*(2,893) = 18.665 (Pillai’s), *p* < 0.001, *n^2^* = 0.043; 3 > 2,1

*Dimensions*					*F*(4,1882) = 40.770 (Pillai’s), *p* < *0.001*, *n^2^* = 0.080
D1. Emotion-focused	2.39(0.33)*	2.41 (0.30)	2.47 (34)	2.43 (0.32)	*F*(2,941) = 12.892 (Pillai’s), *p* < 0.001, *n^2^* = 0.027, 1 > 2 > 3
D2. Problem-focused	2.81 (0.50)	2.85 (0.32)	3.01(0.25)*	2.90 (0.33)	*F*(2,941) = 38.765, *p* < 0.001, *n*^2^ = 0.076; 3 > 2 > 1*

*Emotion-focused strategies (factors)*					*F*(10,1858) = 4.628 (Pillai’s), *p* < 0.001, *n^2^* = 0.036
F1. Avoidant distraction	2.17 (0.49)	2.26 (0.47)	230(0.52)*	2.26 (0.49)	*F*(2,952) = 3.805 (Pillai’s), *p* < 0.05, *n^2^* = *0.008*; 3 > 1
F7. Reducing anxiety	3.00 (0.65)	2.96 (0.58)	3.11(0.71)*	3.02 (0.64)	*F*(2,952) = 4.161, *p* < 0.001, *n^2^* = 0.016, 1,2 < 3
F8. Preparing for the worst	2.61 (0.49)	2.67 (0.46)	2.71 (0.48)	2.67 (0.47)	*F*(2,952) = 1.919, *p* < 0.147 ns, *n^2^* = 0.004
F9. Emotional venting	1.92 (0.44)	1.92 (0.45)	1.82(0.45)*	1.89 (0.45)	*F*(2,952) = 5.697, *p* < 0.001, *n^2^* = 0.012; 1,2 > 3*
F11. Resigned acceptance	2.06 (0.56)	2.08 (0.51)	2.00 (0.51)	2.05 (0.52)	*F*(2,952) = 2.258, *p* < 0.08, *n^2^* = 0.005

*Problem-focused strategies (factors)*					*F*(10,1858) = 4.628 (Pillai’s), *n*^2^ < 0.001, *n^2^* = 0.036
F2. Seeking help	2.76 (0.72)	2.87 (0.65)	3.09(0.69)*	2.92 (0.69)	*F*(2,932) = 15.283, *p* < 0.001, *n^2^* = 0.032; 1,2 < 3
F5. Self-Instructions	2.96 (0.46)	3.00 (0.41)	3.18(0.40)*	3.05 (0.43)	*F*(2,932) = 20.309, *p* < 0.001, *n^2^* = 0.042, 1,2 < 3*
F10. Positive re-appraisal	2.95 (0.56)	2.99 (0.46)	3.20(0.47)*	3.06 (0.49)	*F*(2,932) = 23.028, *p* < 0.001, *n^2^* = 0.047; 1,2 < 3*
F12. Communicating feelings	2.88 (0.79)	2.98 (0.69)	3.18(0.71)*	3.03 (0.71)	*F*(2,932) = 11.865, *p* < 0.001, *n^2^* = 0.025;1,2 < 3
F13. Alternative reinforcement	2.75 (0.43)	2.79 (0.41)	2.92(0.44)*	2.83 (0.43)	*F*(2,932) = 12.290, *p* < 0.001, *n^2^* = 0.026; 1,2 < 3

*SR*, Self-Regulation; *NR*, Non-regulation; *DR*, Dysregulation; *ER*, External Regulation; *ENR*, External Non-regulation; EDR, External Dysregulation; *Featured effect.

#### Effects of Regulatory Teaching on Stress Coping Strategies

There was a statistically significant effect of the *IV RT* (low–medium–high levels) on *total* Coping Strategies. The statistically significant partial effect of the *IV RT (low–medium levels)* was maintained in the dimensions of *Coping Strategies*. There was a statistically significant partial effect of the *IV SR* (low–medium–high levels) for the two dimensions of *Emotion-focused Coping* and *Problem-focused Coping Strategies*, the latter again showing greater statistical effect.

A statistically significant main effect of the *IV RT* (low–medium–high levels) was noted on the factors of *Emotion-focused Coping Strategies*. Also, the statistically significant partial effect of the *IV RT* (low-medium-high levels) was maintained for *F1* (Avoidant distraction), *F7* (Reducing anxiety and avoidance), *F8* (Preparing for the worst), *F11* (Resigned acceptance) and especially, in the use of strategy *F9* (Emotional venting and isolation) for low levels of RT. Complementarily, a statistically significant main effect of the *IV RT* (low–medium–high levels) was noted in the factors of *Problem-focused Coping Strategies*. Also, the statistically significant partial effect of the *IV SR* (low–medium–high levels) was maintained for *F2* (Seeking help), *F12* (Communicating feelings and social support), *F13* (Seeking alternative reinforcement), and with greater statistical significance for the factors *F5* (Self-instructions) and *F10* (Positive re-appraisal and firmness) for high levels of external regulation (RT). See Table 3.

### Interdependent Complex Effects (3 × 3) of the Levels of Self-Regulation (SR) With Levels of Regulatory Teaching (RT) on Stress Coping Strategies

#### Effect on Total Coping Strategies and Dimensions

The *IV SR* (low–medium–high levels) did not show any significant effect in total *Coping Strategies*, but it did produce a statistically significant main effect on the dimensions or factors of coping stress. The statistically significant partial effect of the *IV SR* (low–medium–high levels) was maintained for the two dimensions of *Emotion-focused Coping Strategies* and *Problem-focused Coping Strategies*.

A statistically significant effect of the *IV RT* (low-medium-high levels) was noted in *total* Coping Strategies. The statistically significant partial effect of *IV RT* (low–medium levels) was maintained in the *dimensions* of *Coping Strategies*. The statistically significant partial effect of the *IV RT* (low–medium–high levels) was maintained for the two dimensions of *Emotion-focused Coping Strategies* and *Problem-focused Coping Strategies*.

#### Effect on Specific Factors of Emotion-Focused Coping Strategies

The *IV SR* (low-medium-high levels) was observed to have a statistically significant main effect on the *Factors of Emotion-focused Coping Strategies.* A statistically significant effect appeared of the *IV RT* (low-medium levels) on the *Factors of Emotion-focused Coping Strategies.* There was no statistically significant effect of the interaction SR × RT.

The statistically significant partial effect of the *IV SR* (low–medium–high levels) was maintained for *F1* (Avoidant distraction), *F7* (Reducing anxiety), *F8* (Preparing for the worst), *F9* (Emotional venting and isolation), and *F11* (Resigned acceptance), where the last three factors have greater statistical significance, for students with a lower level of SR. Complementarily, a statistically significant partial effect of the *IV RT* (low–medium–high levels) was maintained for *F1* (Avoidant distraction), *F7* (Reducing anxiety), *F8* (Preparing for the worst), *F9* (Emotional venting and isolation), and *F11* (Resigned acceptance), the last two factors having greater statistical significance, for students with a lower level of RT. There were no significant interaction effects of SR × RT for coping factors in Emotion-focused Coping Strategies. See Table 4.

**TABLE 4:** Interdependent complex effects (3 × 3) of low-medium-high levels of *Self-Regulation (SR)* with low–medium–high levels of *Regulatory Teaching (RT)* on stress coping strategies (*n* = 797).

***SR***	***Low (n* = *199)***			***Medium (n* = *378)***			***High (n* = *220)***			***Variable Effect***	***F(Pillai’s)***
***RT***	***Low***	***Med***	***High***	***Low***	***Med***	***High***	***Low***	***Med***	***High***		
***n*** =	**48**	**106**	**45**	**72**	**190**	**116**	***2*5**	**78**	**117**		
*Coping Strategies*											
*Total*	2.58 (0.33)	2.66 (0.26)	2.71 (0.27)	2.61 (0.26)	2.62 (0.42)	2.74 (0.27)	2.64 (0.24)	2.64 (0.27)	2.73 (0.28)	SR	*F*(2,788) = 0.321, *p* < 0.725 ns, *n^2^* = 0.001

										RT	*F*(2,788) = 10.660, *p* < 0.001, *n^2^* = 0.026
*Dimensions*										SR	*F*(4,1576) = 23.391, *p* < 0.001, *n*^2^ = 0.056
										RT	*F*(4,1576) = 5.751. *p* < 0.00l, *n^2^* = 0.112
D1. Emotion focus	2.47 (0.36)	2.51 (0.33)	2.56 (0.35)	2.36 (0.34)	2.40 (0.27)	2.51 (0.32)	2.32 (0.25)	2.33 (32)	2.39 (34)	SR	*F*(2,788) = 10.546, *p* < 0.001, *n^2^* = 0.026, 1 > 2 > 3
										RT	*F*(2,788) = 5.079, *p* < 0.01, *n^2^* = 0.013, 1 > 2 > 3
D2. Problem focus	2.68 (0.40)	2.81 (0.32)	2.87 (0.30)	2.86 (0.30)	2.85 (0.31)	2.97 (0.30)	2.97 (0.98)	2.94 (0.32)	3.09 (0.32)	SR*	*F*(2,788) = 17.399, *p* < 0.001, *n^2^* = 0.042, 1 < 2 < 3

										RT*	*F*(2,788) = 10.856, *p* < 0.001, *n^2^* = 0.027; 1 < 2 < 3
*Emotion-focused strategies (factors)*										SR*	*F*(10,1774) = 12,225, *p* < 0.001, *n^2^* = 0.067
										RT	*F*(10,1774) = 3,329, *p* < 0.001, *n*^2^ = 0.018
Fl. Avoidant distr.	2.30 (0.42)	2.33 (0.53)	2.39 (0.52)	2.14 (0.52)	2.25 (0.44)	235 (0.48)	2.06 (0.47)	2.21 (0.43)	2.20 (0.55)	SR	*F*(2,890) = 6.369, *p* < 0.001, *n^2^* = 0.014, 1,2 > 3
										RT	*F*(2,890) = 4.151, *p* < 0.01, *n^2^* = 0.016, 1,2 > 3
F7. Red. Anxiety	3.11 (0.62)	3.08 (0.62)	3.24 (0.64)	2.96 (0.63)	2.96 (0.52)	3.21 (0.65)	2.76 (0.62)	2.86 (0.83)	2.92 (0.74)	SR	*F*(2,890) = 9.019, *p* < 0.001, *n^2^* = 0.016; 1 > 2 > 3
										RT*	*F*(2,890) = 5,279, *p* < 0.001, *n^2^* = 0.012; 1,2 < 3
F8. Prep the worst	2.73 (0.55)	2.82 (0.45)	2.89 (0.47)	2.61 (0.43)	2.65 (0.44)	2.71 (0.48)	239 (0.47)	2.53 (0.46)	2.60 (0.44)	SR*	*F*(2,890) = 21.897, *p* < 0.001, *n^2^* = 0.047; 1 > 2 > 3
										RT	*F*(2,890) = 5,045, *p* < 0.001, *n^2^* = 0.012; 1,2 > 3
F9. Emotional vent	2.10 (0.47)	2.10 (0.47)	2.04 (0.58)	1.88 (0.42)	1.90 (0.43)	1.89 (0.40)	1.71 (0.39)	1.75 (0.38)	1.64 (0.35)	SR*	*F*(2,890) = 37.867, *p* < 0.001, *n^2^* = 0.047; 1 > 2 > 3
										RT	*F*(2,890) = l,511, *p* < *0.213* ns, *n^2^* = 0.003
F11. Resigned acc.	2.28 (0.60)	231 (0.56)	2.27 (0.56)	2.00 (0.52)	2.05 (0.46)	2.06 (0.44)	1.70 (0.44)	1.85 (0.45)	1.77 (0.45)	SR*	*F*(2,890) = 50.666, *p* < 0.001, *n^2^* = 0.102; 1 > 2 > 3

										RT	*F*(2,890) = 0.890, *p* < 0.412 ns, *n^2^* = 0.002
*Problem-focused strategies (factors)*										SR	*F*(10,1750) = 15,664, *p* < *0.001*, *n^2^* = 0.082
										RT	*F*(10,1750) = 2,591, *p* < 0.001, *n^2^* = 0.015
F2. Seeking help	2.57 (0.80)	2.86 (0.71)	3.02 (0.61)	2.84 (0.68)	2.88 (0.63)	3.06 (0.66)	3.06 (0.56)	2.87 (0.61)	3.12 (0.74)	SR	*F*(2,878) = 4,969, *p* < *0.001, n^2^* = 0.011, 1,2 < 3
										RT*	*F*(2,878) = 7.168, *p* < *0.001*, *n^2^* = 0.016, 1,2 < 3
F5. Self-Instructions	2.78 (0.48)	2.87 (0.42)	2.91 (0.43)	3.04 (0.42)	3.01 (0.38)	3.11 (0.57)	3.16 (0.46)	3.18 (0.39)	334 (0.62)	SR*	*F*(2, 878) = 37,992, *p* < 0.001, *n*^2^ = 0.080; 1 < 2 < 3
										RT*	*F*(2, 878) = 6,483, *p* < *0.001*, *n^2^* = *0.015*; 1 < 2 < 3
F10. Re-appraisal	2.65 (0.58)	2.78 (0.49)	2.83 (0.49)	3.05 (0.51)	3.01 (0.41)	3.12 (0.41)	3.22 (0.44)	3.28 (0.38)	3.44 (0.37)	SR*	*F*(2,878) = 69.018, *p* < 0.001, *n^2^* = *0.136*; 1 < 2 < 3
										RT	*F*(2,878) = 6,237, *p* < 0.001, *n^2^* = 0.014; 1,2 < 3
F12. Comm. feelings	2.66 (0.90)	2.94 (0.75)	3.12 (0.74)	3.01 (0.72)	2.96 (0.78)	3.18 (0.71)	3.23 (0.59)	3.04 (0.64)	3.19 (0.74)	SR	*F*(2,878) = 6,896, *p* < 0.001, *n^2^* = 0.015; 1 < 2 < 3
										RT	*F* (2,878) = 5,414, *p* < 0.012, *n^2^* = 0.012; 1 < 2 < 3
F13. Altern. reinforc.	2.67 (0.50)	2.80 (0.38)	2.84 (0.40)	2.79 (0.41)	2.76 (0.4)	2.89 (0.42)	2.82 (0.41)	2.87 (0.46)	2.96 (0.47)	SR	*F*(2,878) = 5.069, *p* < 0.001, *n^2^* = 0.011; 1 < 2 < 3
										RT	*F*(2,878) = 5.069, *p* < 0.001, *n^2^* = 0.011

*Statistical effect with higher *F* value: featured effect.

#### Effect on Specific Factors of Problem-Focused Coping Strategies

A statistically significant main effect of the *IV SR* (low–medium–high levels) was noted on the *Factors of Problem-focused Coping Strategies.* There was a statistically significant effect of the *IV RT* (low-medium-high levels) on the *Factors of Problem-focused Coping Strategies.* There was no significant effect of the SR × RT interaction.

**FIGURE 1:**
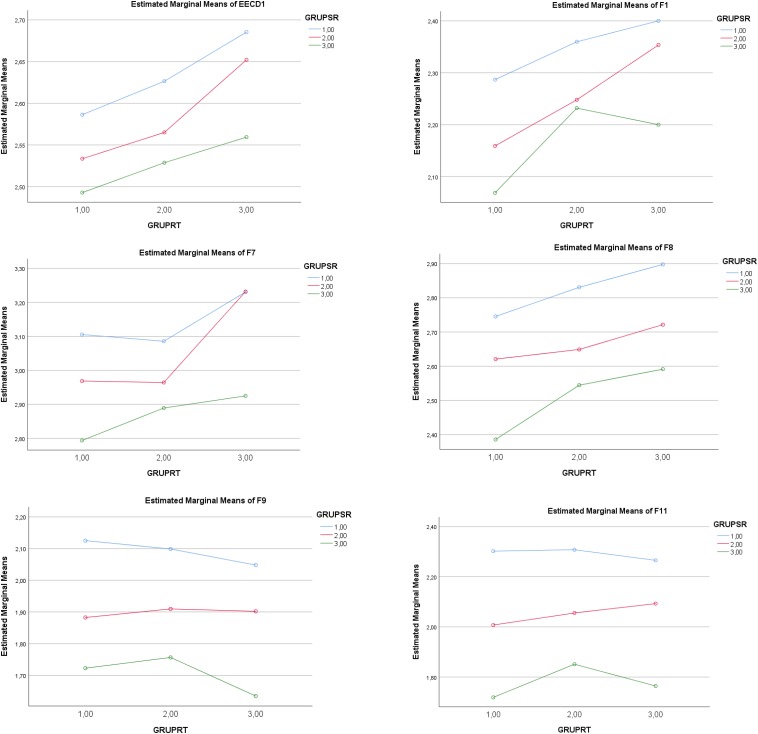
Graphic representation of the effect of low(1)–medium(2)–high(3) levels in the IV *Self- Regulation* (GRUPSR) and low(1)–medium(2)–high (3) levels in the IV *Regulatory Teaching* (GRUPRT) on *Emotion-focused Coping Strategies* (EECD1)*;* EECF1. Avoidant distraction; EECF7. Reducing anxiety; EECF8. Preparing for the worst; EECF9. Emotional venting; EECF11. Resigned acceptance.

**FIGURE 2:**
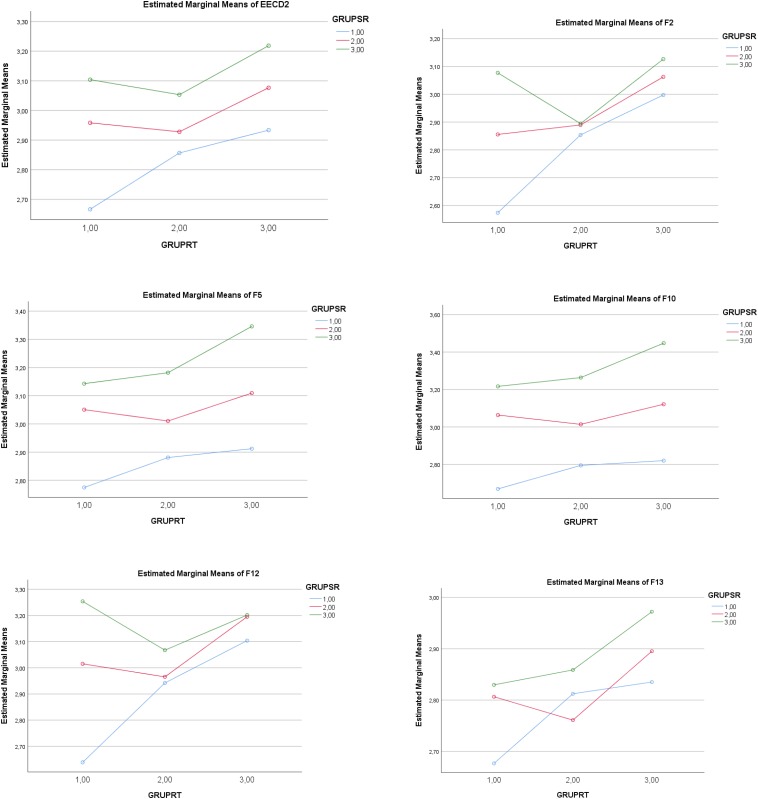
Graphic representation of the effect of low(1)–medium(2)–high(3) levels in the IV *Self-regulation* (GRUPSR) and low(1)–medium(2)–high (3) levels in the IV *Regulatory Teaching* (GRUPRT) on *Problem-focused Coping Strategies*EECF2. Seeking help; EECF5. Self-Instructions; EECF10. Positive re-appraisal; EECF12. Communicating feelings; EECF13. Alternative reinforcement.

The statistically significant partial effect of the *IV SR* (low–medium–high levels) was maintained for *F2* (Seeking help), *F5* (Self-Instructions), *F10* (Positive re-appraisal), *F12* (Communicating feelings and social support), and *F13* (Alternative reinforcement). Complementarily, a statistically significant partial effect of the *IV RT* (low-medium-high levels) was maintained for *F2* (Seeking help), *F5* (Self-Instructions), *F10* (Positive re-appraisal), *F12* (Communicating feelings and social support), and *F13* (Alternative reinforcement). There were no significant interactions of SR × RT for coping factors in the Emotion-focused Coping Strategies. See Table 4 and Figures 1, 2.

### Combination Typology for Understanding Stress Coping Strategies

#### Preliminary Analysis

The MANOVA showed statistically significant differences in the levels of SR and RT variables among the five groups, showing them to be adequately configured according to what is established in Table 4. See the statistical effects in the Table 5.

**TABLE 5:** Effects of combination types on stress coping strategies (*n* = 797).

**DVs**	**Combination Types (IVs)**					
	**1**	**2**	**3**	**4**	**5**	**Effects *post hoc***
	**(*n* = 48)**	**(*n* = 178)**	**(*n* = 260)**	**(*n* = 194)**	**(*n* = 117)**	
*Configuration Group*						*F*(8,2050) = l87.65 (Pillai), *p* < *0.001*, *n^2^* = 0.422
*Self-Regulation*	2.65 (37)	3.02 (0.42)	3.41 (0.44)	3.80 (0.39)	4.23 (0.29)	*F*(1,1029) = 302.61, *p* < 0.001, *n^2^* = 0.302, all *p* < 0.001
*Regulatory Teaching*	2.75 (0.32)	3.26 (0.50)	3.65 (0.68)	4.04 (0.44)	4.39 (0.30)	*F*(1,1029) = 243.64, *p* < 0.001, *n^2^* = 0.614, all *p* < 0.001

*Coping strategies*						
*Total*	2.58 (33)	2.64 (0.26)	2.64 (0.24)	2.70 (0.28)	2.74 (0.29)	*F*(4,792) = 5,046 (Pillai), *p* < 0.001, *n*^2^ = 0.025;5,4 > 3,2,1**

*Dimensions*						*F*(8,1584) = 13.771 (Pillai), *p* < 0.001, *n^2^* = 0.095, *pow* = 1.0
*D1. Emotion focus*	2.47 (0.36)	2.45 (0.34)	2.42 (0.29)	2.44 (0.33)	2.39 (0.34)	*F*(4,792) = 0.856, *p* < 0.490 *ns, n^2^* = 0.275
*D2. Problem focus*	2.68 (0.40)	2.83 (0.31)	2.86 (0.31)	2.96 (0.31)	3.09 (0.32)	*F*(4,792) = 2,107, *p* < 0.001, *n^2^* = 0.093; 5,4 > 3,2 > 1**

*Emotion-focused factors*						*F*(20,3524) = 9,981 (Pillai), *p* < 0.001, *n^2^* = 0.054, pow = 1.0
Fl. Avoidant distrac.	2.29 (0.42)	2.26 (0.54)	2.25 (0.46)	2.29 (0.47)	2.20 (0.55)	*F*(4,882) = 0.808, *p* < 0.523 ns, *n^2^* = 0.004
F7. Reducing anx.	3.12 (0.62)	3.02 (0.62)	2.99 (0.57)	3.07 (0.64)	2.93 (0.75)	*F*(4,882) = 16.056, *p* < 0.001, *n^2^* = 0.069
F8. Preparing worst	2.73 (0.55)	2.73 (0.46)	2.66 (0.47)	2.64 (0.48)	2.60 (0.44)	*F*(4,882) = 1.405, *p* < 0.231 ns, *n^2^* = 0.006
F9. Emotional vent	2.11 (0.47)	2.00 (0.46)	1.90 (0.46)	1.83 (0.40)	1.64 (0.35)	*F*(4,882) = 17.753, *p* < 0.001, *n^2^* = 0.076, 5,4 < 3 < 2,1**
Fll.Resigned accep.	2.29 (0.60)	2.18 (0.56)	2.05 (0.49)	1.99 (0.47)	1.77 (0.45)	*F*(4,882) = 16.319, *p* < 0.001, *n^2^* = 0.070, 5,4 < 3 < 2,1**

*Problem-focused factors*						*F*(20,3524) = 9,981 (Pillai), *p* < 0.001, *n*^2^ = 0.054, pow = l,0
F2. Seeking help	2.57 (0.63)	2.85 (0.70)	2.92 (0.73)	2.99 (0.64)	3.12 (0.73)	*F*(4,882) = 7.644, *p* < 0.001, *n*^2^ = 0.034, 5 > 4,3 > 2,1**
F5. Self-Instructions	2.77 (0.47)	2.94 (0.43)	3.00 (0.40)	3.14 (37)	3.34 (36)	*F*(4,882) = 30,614, *p* < 0.001, *n*^2^ = 0.122, 5 > 4 > 3 > 2,1**
F10. Reappraisal	2.66 (0.57)	2.90 (0.51)	2.99 (0.44)	3.18 (0.40)	3.44 (0.77)	*F*(4,882) = 45.640, *p* < 0.001, *n*^2^ = 0.171 5 > 4 > 3 > 2,1**
F12. Comm feelings	2.63 (0.89)	2.97 (0.74)	3.01 (0.68)	3.14 (0.68)	3.20 (0.74)	*F*(4,882) = 7.587, *p* < 0.05, *n*^2^ = 0.033, 5,4 > 3,2 > 1**
F13 Altern reinforc	2.67 (0.49)	2.81 (0.39)	2.78 (0.40)	2.87 (0.44)	2.97 (0.47)	*F*(4,882) = 7.100, *p* < 0.001, *n*^2^ = 0.31, 5,4,3,2 > 1**

*Type 1* (Low Self-Regulation and Low Regulatory Teaching); *Type 2* (Low Self-Regulation and High Regulatory Teaching); *Type 3* (Medium Self-Regulation and Medium Regulatory Teaching); *Type 4* (High Self-Regulation and Low Regulatory Teaching); *Type 5* (High Self-Regulation and High Regulatory Teaching). For more information, see Table 4. **p < 0.01.

#### Stress Coping Strategies

**FIGURE 3:**
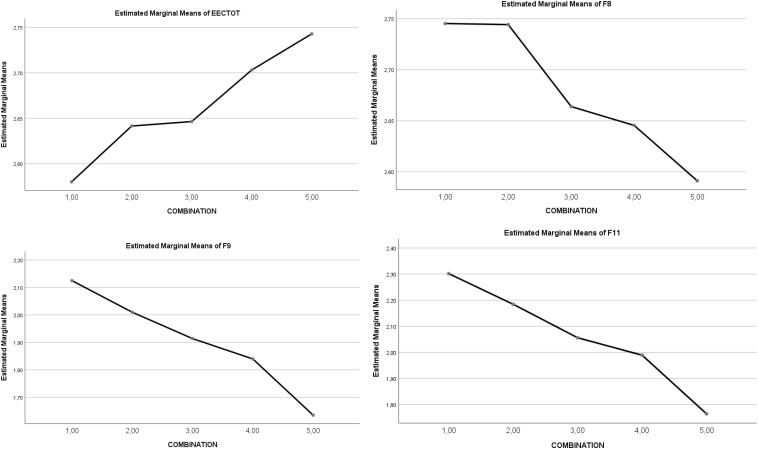
Graphic representation of the effects of the combination types (levels 1–5) on *Emotion-focused Coping Strategies;* EECTOT = Total strategies; F8 = Preparing for the worst; F9 = Emotional venting; F11 = Resigned acceptance.

**FIGURE 4:**
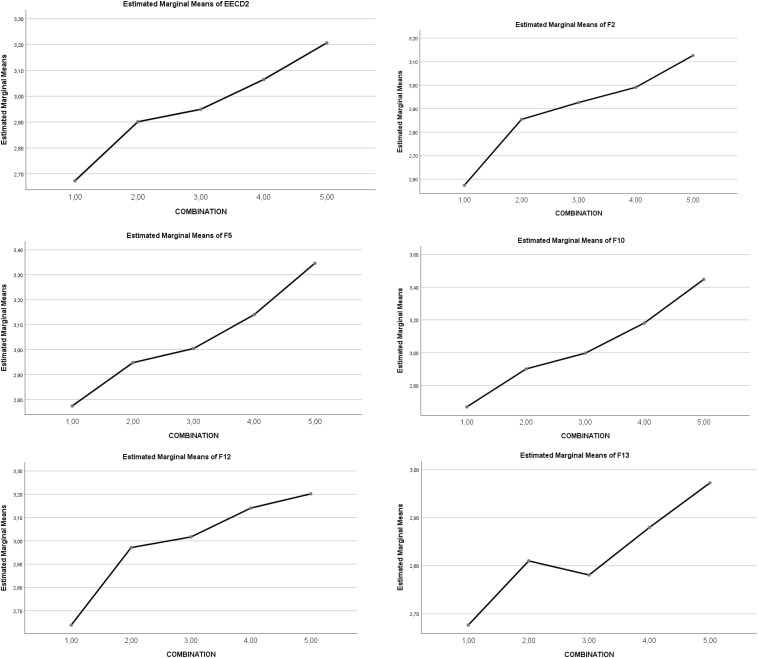
Graphical representation of the effects of the combination types (levels 1–5) on *Problem-focused Coping Strategies.* EECD2 = Problem-focused strategies; F2 = Seeking help; F5 = Self-Instructions; F10 = Reappraisal; F12 = Comm. feelings and social support; F13 = Alternative reinforcement.

There was a statistically significant main effect of the *five IV combinations of SR and RT* on Total Coping strategies [5,4 > 3,2,1]. In the case of *Emotion-focused Coping Strategies*, no statistically significant effect appeared, but in *Problem-focused Coping Strategies* there was a statistically significant effect in favor of high levels [5, 4 > 3 > 2,1]. The statistically significant partial effect was maintained for factors of *Emotion-focused Coping Strategies* (F9. *Emotional venting*, and F11. *Resigned acceptance*), and for the *Problem-focused Coping Strategies* (all factors: 5,4 > 3,2,1). Thus, total coping behaviors progressively increased through the five levels of interaction. Overall, the clearest effects are: higher interaction levels (1–5) leading to a decrease in factors of *Emotion-focused Coping Strategies* (F8, F9, F11), and to an increase in factors of *Problem-focused Coping Strategies* (F2, F5, F10, F12, F13). See Table 5. A graphic representation of the differential progressive effect of combined SR and RT levels is shown in Figures 3, 4.

## Discussion and Conclusion

SRL vs. ERL Theory (de la Fuente, 2017) predicted that university students’ coping strategies could be determined, jointly, by the students’ degree of *self-regulation* and by the level of contextual, *external regulation* from the teaching process. Furthermore, this type of interaction could be understood as the combination of *low-medium-high levels* of the two factors, as supported by prior evidence in this direction (de la Fuente et al., 2015a, 2017b).

For *hypotheses 1 and 2*, the results offer evidence that a (1) *graded increase in level of regulation* (internal and external) gives rise to a proportionate decrease in emotion-focused strategies, and a proportionate increase in problem-focused coping strategies. By contrast, a (2) *graded decrease in level of regulation* (internal and external) gives rise to a proportionate increase in emotion-focused strategies, and a proportionate decrease in problem-focused coping strategies. The hypothesis can be considered partially validated.

Analysis of the *simple effect* of the variables showed that level of SR positively determined the level of problem-focused strategies and negatively determined the level of emotion-focused strategies. Likewise, the level of RT showed a similar trend. This result is consistent with prior evidence from this line of research (de la Fuente et al., 2017b), as well as from other studies (Holinka, 2015; Collie et al., 2019). On the other hand, analysis of the *combined effect* of the variables showed two independent main effects, both from SR and from RT, but did not show a crossover interaction, consistently with previous evidence on the effect of these two variables on coping strategies (de la Fuente et al., 2017b, 2019). Finally, when analyzing a graded increase in the *combination level (scale of 1–5)*, the results are very consistent with the idea that the combination of the two types of regulation (person × context) significantly predicts a decrease in emotion-focused strategies and an increase in problem-focused strategies. These results are very consistent with others that our research team has recently found and reported (de la Fuente et al., 2019, p. 14), where positive achievement emotions were found to increase with higher ranking combinations of internal (SR) and external (RT) regulation, and negative emotions increased with lower ranking combinations of internal (SR) and external (RT) regulation. Consequently, the coping behaviors analyzed here –as a variable of emotional or meta-emotional regulation—would reflect a similar response schema for managing each type of emotionality, according to the degree of SR and RT. Nonetheless, based on results from the two studies, it is not yet possible to establish a causality relationship for types of achievement emotions or coping strategies, an aspect which remains for further empirical analyses.

### Theoretical Implications

These findings are important for this theoretical model because they lend support to the premise that both the student’s lack of regulation and a lack of regulation in teaching tend toward negative emotionality, and consequently, to greater use of emotion-focused strategies, to the detriment of problem-focused strategies. By contrast, higher levels of regulation in the student and higher levels of RT both contribute to positive emotionality, tending toward a greater use of problem-focused strategies, given that emotion-focused strategies for managing negative emotionality are not needed. This supports the importance of university students’ perception of the teaching process (Aldrupa et al., 2019). These tendencies are similar to those found in other studies (de la Fuente et al., 2017b, 2019), lending empirical support to the assumption that the *combination* of individual and contextual regulation characteristics delimits the level of stress, just as is predicted by SRL vs. ERL Theory (de la Fuente, 2017). In other words, students with a lower level of SR (non-regulation or dysregulation), who are exposed to non-RT processes (no external regulation or dysregulating), are the ones who produce the greatest stress factors and show the greatest symptomology of stress (de la Fuente et al., 2020; in review), leading to greater application of emotion-focused strategies and to reduced focus on the problem. The opposite occurs in the case of students with high SR who are exposed to highly RT.

This theoretical contribution allows us to progress to a broader view of the *Theory of Self-Regulated Learning* (Zimmerman, 2001, 2008; Zimmerman and Labuhn, 2012). We can infer that the context -in this case the presence or lack of *effective teaching-* may have an active regulatory role, promoting and aiding the student’s SR, and becoming just as important as the university student’s own SR for predicting emotional behaviors of learning and ways of coping. It also enables us to operationalize the concept of *Self-regulation vs. External-Regulation* (Vermunt, 2005, 2007; Vermunt and Donche, 2017; and further specified by Vanthournout et al., 2014), since external regulation is conceptualized not as the opposite of internal, self-regulation, but as something that fosters SR, thereby resolving certain recent criticisms (Hederich-Martínez and Camargo, 2019).

The coping strategy labeled F9 (*emotional venting and isolation*) requires special attention. It is plausible that this dysregulatory behavior is a link between students’ *learning and achievement problems* and certain *health problems -*alcohol intake, substance abuse or behavioral excesses (Freire et al., 2016; Garzón-Umerenkova et al., 2018; Kamijo and Yukawa, 2018). In other words, although the causes of learning and achievement problems can be both internal to the student (cognitive, meta-cognitive, motivational or meta-motivational in origin) and external, in the teaching process (its adjustment or maladjustment), what is certain is that the meta-emotional factors addressed here are significant in health predictions. Prior evidence has shown that negative emotionality, lack of confidence and lack of resilience correlate positively to the *surface approach* and negatively to the *deep approach* (de la Fuente et al., 2017a). It is therefore necessary to take this combination into account in the prevention of stress factors in university teaching-learning processes (Palmer and Rodger, 2009; Alonso-Tapia et al., 2018).

### Limitations and Future Directions

The present research study has several limitations worth mentioning. The sample should be improved by adding university students from different degree programs. The degree to which stress factors are determined by the student’s personality variables (presage) also remains to be verified, as well as the connection between such variables and variables that explain good learning (as a process) and academic achievement (as a product). Other studies from our research team have already reported the importance of achievement emotions in different situations –in class, study time, testing (de la Fuente et al., 2019)—and upcoming studies will address these complex relations.

One especially important aspect for future investigation is the relationship of levels of self-regulation and external regulation to the concept of *flexible emotion regulation* (Gross, 2008, 2014, 2015a,b), with its recent important contributions (Kobyliñska and Kusev, 2019), and the coping strategies associated with each combination type. It would also be desirable to evaluate RT produced by university teachers as a function of their own emotions, given that some relationships have already been found (Frenzel et al., 2016, 2018). Another important aspect to be studied is the cross-cultural validity of these results, recognizing our limitation to a Spanish-speaking environment, and the need to expand this evidence to English-speaking samples, as well as other international groups, something to be addressed in future research. Special attention should also be given to gender differences, not analyzed in the present research study, but where important effects can be found, as shown by one recent study (Cabanach et al., 2009; Martínez et al., 2019).

### Implications for the Practice of Educational Psychology at University

Applied implications from this research refer to two aspects. On one hand, students must be trained in the importance of self-regulating behavior when learning at university, not only in its *meta-cognitive* aspects (deep vs. surface learning approaches), but also in the relevance of *emotional factors* (achievement emotions), *meta-emotional factors* (emotion-focused vs. problem-focused coping strategies) and *meta-behavioral factors* (behavioral SR). On the other hand, it is essential that university teachers be trained to minimize stress factors through the design of their teaching process. The concept of *effective teaching* is associated with well-planned teaching, and with fostering in students a perception of control (Paris and Winograd, 2003; Putwain et al., 2019; Shannon et al., 2019). If students with a low level of SR perceive more stress factors and also experience more stress symptomology inherent to the teaching process, any innovative teaching design should take this personal factor into account.

When implementing innovations in the university teaching process, it is important to consider what type of context is being designed, within the framework of the *SRL vs. ERL Theory* (de la Fuente, 2017). If the context is non-regulating or dysregulating, it will probably not help students improve their learning process, especially if students have low SR. As seen in prior evidence, students with little SR require greater external regulation. Certain prior evidence has shown results consistent with this idea (Shaw et al., 2017; Bingen et al., 2019; Kassymova et al., 2019).

## Data Availability Statement

The datasets analyzed in this manuscript are not publicly available. Requests to access the datasets should be directed to jdlfuente@unav.es.

## Ethics Statement

The studies involving human participants were reviewed and approved by the Comité de Ética, Universidad de Navarra. The patients/participants provided their written informed consent to participate in this study.

## Author Contributions

JF have made the general analysis and first version of the manuscript. MG-T has made the second review of the Self-regulation and Externally regulation sections of Coping Stress. JA and JG-T have performed a review of the data analysis. RA and SF have participated in the data collection.

## Conflict of Interest

The authors declare that the research was conducted in the absence of any commercial or financial relationships that could be construed as a potential conflict of interest.
